# Understanding paleo-earthquakes in the Kuril Trench based on Late-Holocene tsunami deposits in the distal region from wave sources, northern Hidaka, Hokkaido, Japan

**DOI:** 10.1371/journal.pone.0298720

**Published:** 2024-04-17

**Authors:** Ryo Nakanishi, Juichiro Ashi, Satoshi Okamura, Yusuke Yokoyama, Yosuke Miyairi

**Affiliations:** 1 Graduate School of Science, Kyoto University, Kyoto, Kyoto, Japan; 2 Atmosphere and Ocean Research Institute, The University of Tokyo, Kashiwa, Chiba, Japan; 3 Graduate School of Frontier Science, The University of Tokyo, Kashiwa, Chiba, Japan; 4 Hokkaido University of Education, Sapporo, Hokkaido, Japan; 5 Hokkaido Soil Research Co-operation, Sapporo, Hokkaido, Japan; 6 Department of Earth and Planetary Sciences, Graduate School of Science, The University of Tokyo, Bunkyo-ku, Tokyo, Japan; 7 Graduate Program on Environmental Science, Graduate School of Arts and Sciences, The University of Tokyo, Bunkyo-ku, Tokyo, Japan; 8 Japan Agency for Marine-Earth Science and Technology (JAMSTEC), Yokosuka, Kanagawa, Japan; 9 Research School of Physics, The Australian National University, Canberra, Australia; Rikkyo University, JAPAN

## Abstract

Geological evidence, such as tsunami deposits, is crucial for studying the largest rupture zone of the Kuril Trench in Hokkaido, Japan, due to its poor historical record. Although 17th-century tsunami deposits are widely distributed across Hokkaido, the presence of multiple wave sources during that period, including the collapse of Mt. Komagatake, complicates the correlation with their wave sources. Understanding the regional distribution of these tsunami deposits can provide valuable data to estimate the magnitude of megathrust earthquakes in the Kuril Trench. The northern part of Hidaka, Hokkaido, where tsunamis from multiple wave sources are expected to overlap, is distant from the Kuril Trench. To clarify the depositional history of tsunami deposits in such distal areas, evaluating the influence of the depositional environments on the event layer preservation becomes even more critical. We conducted field surveys in Kabari, located in the northern Hidaka region, identifying three sand layers from the 10th to the 17th century and two layers dating beyond 2.3 thousand years ago. The depositional ages of most sand layers potentially correlate with tsunami deposits resulting from the Kuril Trench earthquakes. Utilizing reconstructed paleo-sea level data, we estimated that most sand layers reached approximately 2 m in height. However, it is noteworthy that the latest sand layer from the 17th century exhibited an unusual distribution, more than 3 m in height. This suggests a different wave source as the Mt. Komagatake collapse. The discovery of multiple sand layers and their distributions is crucial to constraining the maximum magnitude of giant earthquakes in the Kuril Trench and understanding the volcanic tsunami events related to Mt. Komagatake.

## Introduction

Tsunami deposits play a crucial role in understanding the magnitude and history of paleo-tsunamis that predate historical records. Moreover, the distribution of tsunami deposits is related to the extent of tsunami inundation, making them a valuable tool for estimating earthquake magnitudes [1–5]. In particular, tsunamis resulting from megathrust earthquakes leave deposits that extend broad areas. Therefore, conducting comprehensive geological investigations in regions far from the wave source becomes essential [6]. This contributes not only to the precise estimation of tsunami magnitudes and, ultimately, earthquake magnitudes for disaster prevention, such as creating more accurate hazard maps but also to understanding tsunami deposits in distal areas where few studies have been conducted.

**Fig 1:**
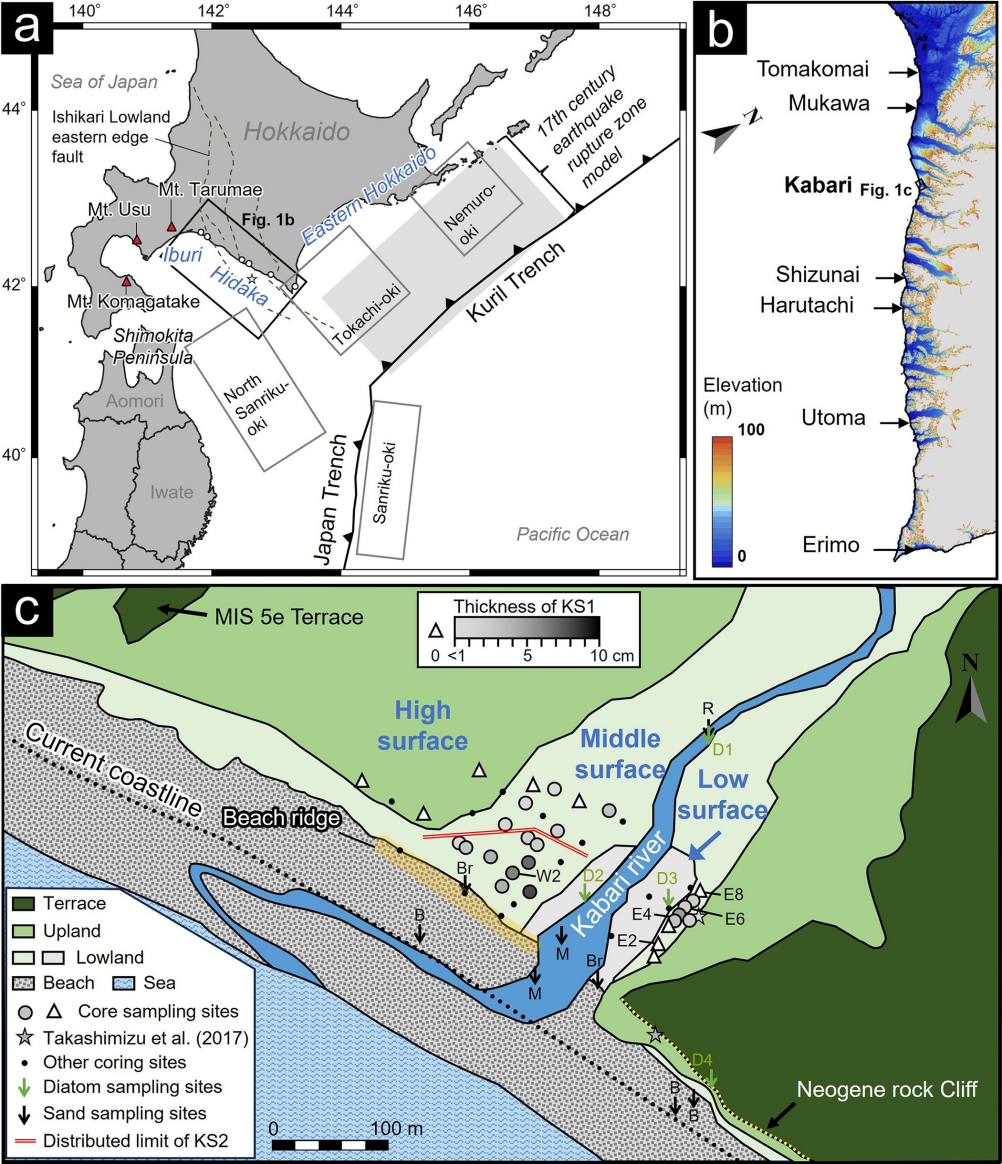
Maps of the study area. (a) Overall view and earthquake rupture zone. Triangles indicate volcanoes that are the source of tephras. The solid gray box and the gray shading indicate the rupture zones of the historical and reconstructed paleo-earthquakes, respectively. The dotted lines show the distribution of active faults. The star indicates the epicenter of the 1982 Urakawa-oki earthquake. The solid black box marks the range shown in Fig 1B. (b) DEM data for coastal areas (Geospatial Information Authority of Japan: https://fgd.gsi.go.jp/download/menu.php) and tsunami deposit study sites. (c) Topographic classifications and coring sites observed tephra layers in the Kabari area. The circles with grayscale indicate the layer thickness of Sand KS1. Triangles indicate sites where Sand KS1 was not identified. Stars indicate coring sites reported by Takashimizu et al. [11]. The red double line shows the distribution limits of Sand KS2. Black and green arrows indicate sites where modern reference samples of the grain size and diatom analysis were collected, respectively. The reference sample legends are B: beach, R: river, Br: beach ridge, M: river mouth, and D1–D4 corresponding to the diatom samples shown in Table 2.

The Kuril Trench is a subduction zone where large earthquakes of Mw ~8 have occurred at recurring intervals, typically spanning several decades [7] (Fig 1A). Additionally, geological records in eastern Hokkaido suggest giant earthquakes (here we mean Mw ~9) with longer recurrence intervals [2, 8, 9]. Particularly, tsunami deposits in the 17th century have been reported along the eastern Hokkaido coast, directly facing the Kuril Trench [2, 8, 9]. Nevertheless, it remains uncertain how far this tsunami has extended from the Kuril Trench. Importantly, the fault model in the 17th century has been utilized as the reference event for the largest potential earthquake scenario of the Kuril Trench when developing hazard maps [10]. Consequently, achieving an accurate reconstruction of its rupture zone is a pivotal factor in disaster prevention efforts within this region.

The Hidaka to Iburi regions, which do not directly face the trench, have reported the presence of tsunami deposits below the volcanic ashes in the 17th century [11–15]. According to historical documents, the 17th-century tsunami deposits in these distant areas include several potential wave sources in addition to earthquakes in the Kuril Trench, such as the 1640 CE Mt. Komagatake collapse and the 1611 CE Keicho tsunami [15]. The 1611 CE Keicho tsunami is considered to have originated in Sanriku-Oki because of its high wave height along the coast of Iwate Prefecture [16]. A historical document called "Matsumae Kaki" mentioned that many casualties have occurred in Hokkaido, but the details are not clear. The collapse following the 1640 CE eruption of Mt. Komagatake caused many casualties from the high tsunami around this volcano [17], and it has been modeled that the tsunami reached as far as the Iburi region [12]. The estimated magnitudes of paleo-earthquakes depend significantly on whether these tsunami deposits are from those of the megathrust earthquake in the Kuril Trench. The magnitudes of the earthquake are expected to be Mw 8.8 and Mw >9.0, respectively, depending on whether the correlation of the tsunami deposits extends to the eastern Hokkaido or Iburi coast [1, 2, 10, 18]. Therefore, the 17th-century tsunami deposits in the Iburi and Hidaka coasts need to be carefully compared with the tsunami deposits in eastern Hokkaido because of the existence of the Komagatake tsunami in western Hokkaido.

For tsunamis occurring before the 17th century, the estimated average recurrence intervals in the Kuril Trench have been around 400 years, but they have displayed variations spanning several hundred years [2, 9, 19]. While multiple sand layers have been observed from eastern Hokkaido to central Hidaka (refer to [20] for a detailed chronology) [2, 8, 9, 19–22], such evidence has not yet been confirmed in the northern Hidaka area (Fig 1A) [11]. The northern Hidaka area is expected to be a region where the westward Komagatake tsunami and the tsunami caused by a megathrust earthquake might overlap. Therefore, for a more precise reconstruction of the maximum rupture zone associated with giant earthquakes in the Kuril Trench, it is important to carefully compare regional tsunami deposits and conduct a comprehensive distribution survey over an area distal from the Kuril Trench.

In regions distant from the trenches, even tsunamis caused by giant earthquakes exhibit smaller wave heights than those observed in the near field, and their traces are preserved within a limited area along the coastline. To reveal a formation factor and a distribution associated with wave heights of event layers, it is necessary to reconstruct relative sea-level changes. Given that the preservation of thin event layers is influenced by coastal depositional environments, a thorough discussion based on the depositional context is essential for reconstructing tsunami history [22].

In the Kabari area of northern Hidaka, Hokkaido, Japan, tsunami deposits around the 17th century have been previously reported (Fig 1B) [11]. Here, we present findings on coastal depositional environments, offering a more detailed insight into the distribution of tsunami deposits and older event layers. Additionally, we estimate the paleo-sea level during past tsunami events by reconstructing the depositional context to assess the preservation and distribution of sand layers. The distribution of these tsunami deposits holds significant importance for numerical modeling aimed at estimating the magnitude of tsunamis from the giant earthquakes in the Kuril Trench and the collapse of Mt. Komagatake. Furthermore, the stratigraphy and dating of these tsunami deposits provide valuable indicators of tsunami histories in the northern Hidaka region, situated in a distal area from trenches.

### Observed earthquakes and previous tsunami deposit studies

In the vicinity of the Kabari area, tsunamis from Mw ~8 earthquakes have been observed with maximum tsunami run-up heights reaching approximately 2 m along the coastal area [21]. Specifically, these events include the 2003 Tokachi-oki (Mw 8.1), 1968 North Sanriku-oki (Mw 8.1), and 1933 Sanriku-oki (Mw 7.9) earthquakes [7] (Fig 1A). Regarding active faults, the 1982 Urakawa-oki earthquake (Mw 6.9) occurred on the seaward extension of thrust faults within the Ishikari Lowland eastern edge fault system [23], resulting in tsunami heights of less than 1 meter along the Hidaka coast [24].

Takashimizu et al. [11] reported a sand layer around the 17th century in Kabari (Fig 1C). This layer was identified as a tsunami deposit from its thinning and fining inland and the presence of diatom valves originating from the seaward. The sand layer was distributed from the present shoreline to 150 m inland, situated at an elevation of 3.86 m above the current sea level (asl).

For the central to southern Hidaka region, several tsunami deposit layers with recurrence intervals spanning several hundred years have been discovered despite the limited preservation periods for these deposits [20–22]. Radiocarbon dating of these tsunami deposits has indicated that most of these sand layers can correlate with tsunami deposits found in eastern Hokkaido. The wave source for the 17th-century tsunami deposits in Shizunai, central Hidaka, was estimated through sediment transport modeling, revealing that only the Mw 8.8 model within the Kuril Trench could accurately replicate the distribution of the sand layer [25].

Tsunami deposits have been found as high as 8 m asl in Mukawa [15], situated northwest of Kabari. A numerical simulation of the 1640 CE Komagatake tsunami roughly reproduced the distribution of tsunami deposits in Mukawa [12]. This simulation calculated a wave height of approximately 4 m along the Kabari area’s coastline. In contrast, a tsunami simulation based on the Kuril Trench model (Mw 8.8) indicated wave heights of approximately 2 m in Kahari [2].

### Study area and geological settings

The Kabari area, located in the northern part of Hidaka, is situated approximately 260 km away from the Kuril Trench (Fig 1). In this area, we find the development of marine and river terraces, along with the partial formation of wetlands in the lowland areas. The coastal zone comprises sandy beaches and back-barrier wetlands intersected by the Kabari River. Despite its relatively flat topography, one can observe a series of beach ridges that run between the sandy beach and wetlands. These marine terraces are categorized into four elevations, each corresponding to specific marine isotope stages (MIS) 5e, 7, 9, and 11 [26] (S1 Fig). During MIS 5e, the marine terraces reach heights of around 55 m, whereas during MIS 7, they rise to approximately 90 m. The height of these marine terraces gradually decreases as one moves westward [27] (S1 Fig), indicative of a westward tilt. On the eastern side of the Kabari River, these terraces are composed of Neogene sedimentary rocks, such as sandstone and tuffaceous siltstone [27], which have been extensively exposed due to marine erosion and are covered by beach sand. In the vicinity, fluvial terraces at elevations of less than 10 m asl have developed, roughly categorized into low, middle, and high surfaces (2 m, 3–4 m, and 6–7 m). The northern Hidaka region has experienced significant beach erosion since the 1950s, primarily attributed to reduced sand supply resulting from harbor and embankment construction [28]. A comparison between an aerial photograph from 1944 and the present topography reveals that the coastline has receded by approximately 100 m seaward (S1 Fig).

Based on the glacial isostatic adjustment (GIA) model for MIS 5e, relative sea-level changes in Kabari have been estimated to range from 4.5 to 14 m [29]. Additionally, tide test records at the Tomakomai East Port (recorded from 2016 to 2018) indicate measurements of + 28 cm for mean high water, + 45 cm for mean higher high water, and + 65 cm for the highest astronomical tide.

## Materials and methods

### Field survey

We collected samples using hand borings in both the west and east areas intersected by the Kabari River (Fig 1C). The field survey was conducted with the explicit permission of the landowner. To obtain these samples, we employed the use of a Handy Geoslicer (width: 7 cm; length: 1 m) [30] or a hand corer with a 7 cm diameter. On-site visual descriptions of core samples were conducted for color, grain size, thickness, and sedimentary structure. In total, core samples were acquired from 30 coring sites, predominantly focusing on the middle surface. Additionally, we gathered sand samples from various locations, including beaches, rivers, beach ridges, and river mouths, as potential sources. To precisely determine the coordinates and elevations of these coring sites, we utilized a global navigation satellite system with an error margin of less than 1 cm. This system incorporated multi-band receivers (ZED-F9P U-blox) and relied on a comparison of continuous observation data from the Geospatial Information Authority of Japan’s electronic reference points stationed at the Shizunai station.

### X-ray computed tomography scan

We performed X-ray computed tomography (CT) scans to characterize the internal structure of the core samples caused by differences in density. The samples were imaged using a medical CT scanner (Aquilion PRIME Focus Edition, Canon Medical Systems Corporation) at the Kochi Core Center. The slice and single collimation widths for acquiring CT image data were 0.5 mm. The CT values were expressed as the mode value per slice to indicate vertical fluctuations in the relative density of the cores.

### Grain-size analysis

Grain-size analyses were conducted on sand layers and potential source sands to determine the origins of sand layers. To prepare the sand layers from the core samples, bulk samples underwent pretreatment with hydrogen peroxide to disperse and decompose organic matter. Subsequently, the treated samples were dried and sieved at ½ phi intervals ranging from 4.5 to –2.0 phi, and their weights were recorded. Descriptive statistical values for the measured grain-size distributions, including parameters such as the mean (Mz) and standard deviation (σ_I_), were calculated using the method outlined by Folk and Ward [31]. We also examined vertical changes in the grain size of sand layers at 1 cm intervals.

### Diatom analysis

A diatom assemblage analysis was conducted to reconstruct the depositional environment, such as its hydraulic conditions and salinity, as sea level index points (SLIPs). Salinity was determined qualitatively based on the indicator species [32–35].

Subsamples of 1 cm thickness were collected from the core samples (Site W2 and E1–8) at intervals of several centimeters. The core samples and potential source samples, including river sand, river mouth mud, and low surface mud, underwent treatment with 15% hydrogen peroxide to remove organic matter. Subsequently, 10 cc of each sample was placed onto a microscope slide and sealed with Pleurax medium (Mountmedia, Wako). The slides were observed using an optical microscope (1,000× magnification) and counting continued until 300 diatom valves were observed. Species identification was based on catalogs both from global [36–40] and Japanese references [33, 41–43]. For the classification of diatom species, we considered factors such as salinity (ranging from marine, marine–brackish, brackish, brackish–freshwater, to freshwater) and life form (comprising planktonic, epontic, and benthic categories), drawing on ecological references [32, 35, 44–46]. Diatoms from the Neogene period along the Hidaka coast, as reported by Sagayama et al. [47], were excluded from the assemblage analysis due to their allochthonous contribution linked to the erosion of older deposits [48]. Moreover, we exclusively included species constituting more than 3% of the total, considering them significantly present for the subsequent diatom assemblage analysis, while disregarding less common species.

### Radiocarbon dating

We pretreated selected materials (seeds, charcoal, and plant fragments) as well as bulk peat samples from the core samples with 1 M HCl for 1 hour to eliminate calcium carbonate for radiocarbon dating. After heating the samples, we recovered the resulting CO_2_ and graphitized it using Fe powder in a hydrogen atmosphere [49]. Radiocarbon dating was carried out using a single-stage accelerator mass spectrometer at the Atmosphere and Ocean Research Institute, University of Tokyo [50]. To convert the obtained ^14^C ages into calendar ages, we used OxCal v4.4 [51] in conjunction with the IntCal20 dataset [52]. For calibration, we applied the P_Sequence and Sequence model in OxCal, considering the stratigraphic order [53, 54]. In our age-depth model, we considered the difference between the thickness of the peat layer and the thickness of the sand layer, assuming instantaneous deposition of the sand layers.

## Results

### Stratigraphy

**Fig 2:**
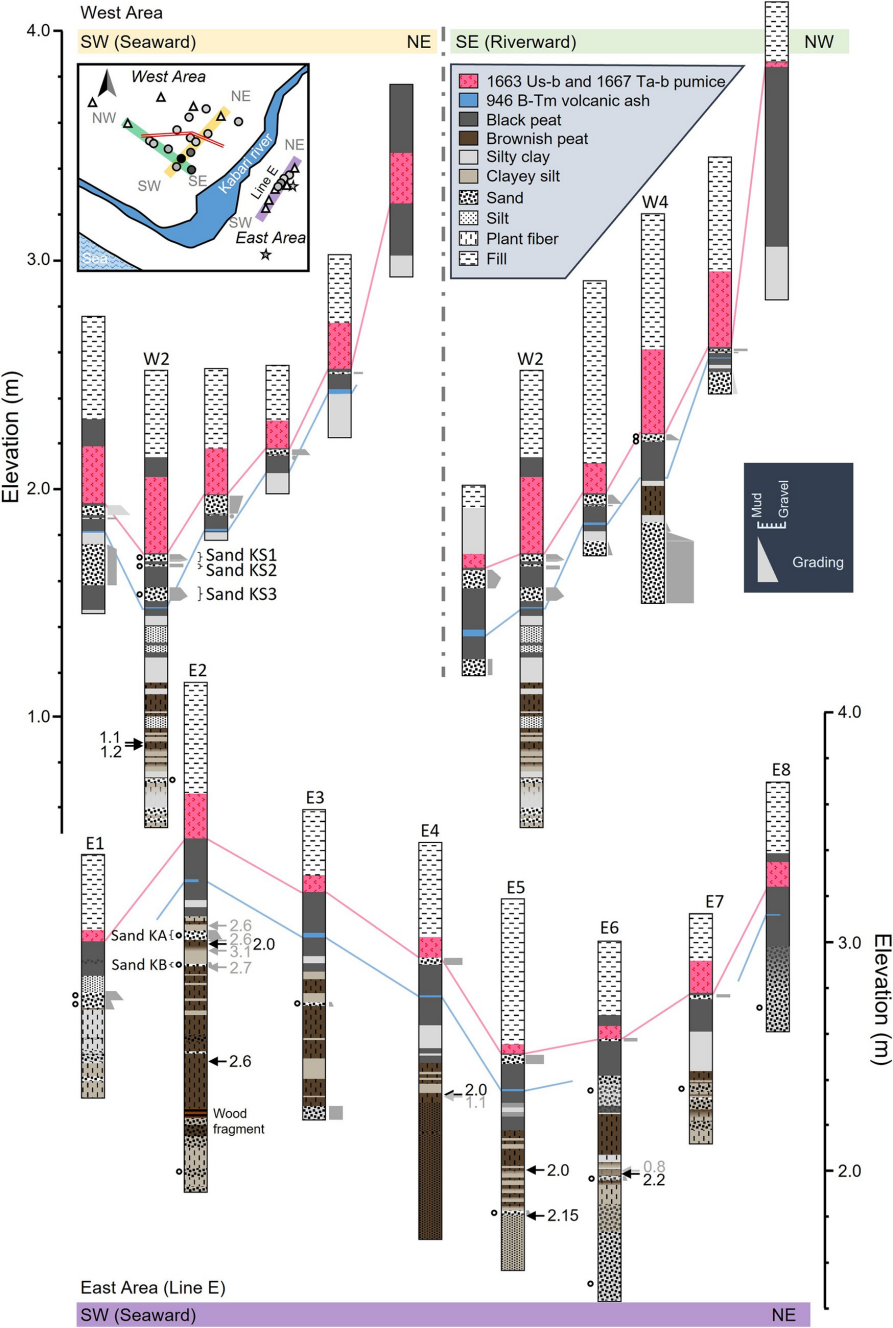
Geological columnar sections. Arrows indicate calibrated radiocarbon dates (ka). The gray numbers are the dates using the bulk sample. Circles indicate sand layers targeted for grain size analysis. The inserted map is the same as Fig 1C and shows the location of the boring sites and survey lines.

This section describes the basic stratigraphy of the Kabari area for the identification of event layers and reconstruction of depositional environments (Fig 2). The sources of the tephra layers in this area have been identified by microscopic observation and chemical composition [14], and this study followed Nakanishi et al. [14]. The low surface sediments comprised silt without a tephra layer. The middle surface sediments comprised silty or clayey sand (e.g., <1.8 m asl in E2), fibrous brownish peat (e.g., 1.8–3.1 m asl in E2), and black peat (e.g., >3.1 m asl in E2) from the bottom to the top. The black peat was interbedded with 946 CE Baegdusan volcano-Tomakomai tephra (B-Tm) of 1–2 cm thickness and 17th-century pumice layers with a thickness of 3–30 cm. The 17th-century pumice layers included 1667 CE Tarumai volcano-b pumice (Ta-b) and 1663 CE Usu volcano-b pumice (Us-b) layers. The fibrous brownish peat frequently interbedded with silt and clay layers. The gravel bed was identified on the seaward side (Fig 1C).

In the west area of the Kabari River, we identified three distinct sand layers within the black peat, situated between the Us-b and B-Tm tephras. These sand layers have been designated as Sand KS1, KS2, and KS3, starting from the top (Fig 2). Below the B-Tm tephra, we observed muddy deposits, sand, and gravel layers. The core extracted from Site W2 exhibited a transition sequence, starting with alternating layers of fibrous brownish peat and sand, gradually shifting to alternating layers of fibrous peat and inorganic silt to clay when moving from bottom to top. The high surface sediments consisted primarily of black peat, with only 17th-century pumice layers and no sand layers.

In the east area, we also detected the presence of the 17th-century and B-Tm tephras within the black peat. Moreover, one sand layer was identified below the Us-b layer, extending up to an elevation of 3.0 m asl (Fig 2). From the fibrous brownish peat layer, we observed one or two sand layers having clear contact with the underlying peat layer, transitioning upward to silt. These sand layers have been named Sands KA and KB, respectively (Fig 2). Additionally, we noted a continuous grayish-white silt layer with several centimeters in thickness within the black peat (e.g., 3.1 m asl in E2). The fibrous brownish peat was interbedded with several yellowish-gray mud layers, eventually transitioning into peat in the upper part (e.g., 1.8–2.0 m asl in E5).

At Sites E1 to E3, closer to the sea, the peat layer transitioned into sandy silt at the lowermost levels. Sites E4 to E6 exhibited a transformation from organic silts to inorganic silts in the lower sections. In contrast, at the inland Sites E7 and E8, a fibrous peat layer was absent, and the lower section featured a sandy layer.

Bulk samples or selected samples such as seeds and charcoal were used for the radiocarbon dating of event layers and SLIPs. The ages of the organic layers obtained by the radiocarbon dating were 1.2 ka in the west area and 2.6 ka in the east area for the lowest stratigraphic level in the measured samples (Table 1 and Fig 2). In the east area, the organic layers have been deposited below the stratigraphic level from which the samples were taken; therefore, the geological record was ~3000 years. At Site W2, charcoals contained in the peat below the Us-b pumice were used for dating. The ^14^C ages of the charcoals differed by 200 years. The younger age result was used because it had a small measurement error and was stratigraphically consistent with the depositional ages of the Us-b and B-Tm tephras. No large plant material or charcoal was found in the peat above the B-Tm tephra, and some bulk samples were used (Table 1).

**Table 1:** Results of radiocarbon dating.

		Depth				Calibrated age (BP)					Modelled age (BP)				Modelled age		
Sample name	Site	(cm)	Material	^14^C age	Error	1σ		2σ		Mean	1σ		2σ		2σ (CE)		Lab number
Sand KS1	W2	81–84									356	292	435	286	1516	1664	
Sand KS1_L1	W2	85	charcoal	587	28	630	547	647	539	595							YAUT-071626
Sand KS1_L2	W2	85	charcoal	387	26	497	335	505	324	434	468	334	491	327	1460	1623	YAUT-079832
Sand KS2	W2	86–88									500	427	511	371	1439	1579	
Sand KS2_L	W2	88	charcoal	436	27	515	487	528	460	494	516	491	526	474	1425	1477	YAUT-073636
Sand KS3_U	W2	93	Bulk peat	970	25	921	802	928	793	858	916	807	926	794	1025	1156	YAUT-079829
Sand KS3	W2	94–100									960	883	994	845	956	1106	
Sand KS3_L	W2	101	Bulk peat	1157	29	1174	977	1178	963	1055	1000	973	1007	960	944	991	YAUT-079831
W2_160cm	W2	160	Reed	1181	24	1174	1062	1179	1002	1103							YAUT-073637
W2_164cm	W2	164	Seed	1297	25	1280	1179	1288	1176	1229							YAUT-079833
E2_106cm	E2	106	Bulk peat	2500	24	2714	2504	2726	2489	2596							YAUT-053036
E2_114cm	E2	114	Bulk peat	2488	25	2706	2495	2721	2470	2590							YAUT-053037
E2_115cm	E2	115	Reed	2056	25	2051	1945	2102	1934	2008							YAUT-079836
E2_119cm	E2	119	Bulk peat	2977	26	3207	3079	3229	3065	3144							YAUT-053038
E2_124cm	E2	124	Bulk peat	2588	30	2752	2727	2769	2542	2727							YAUT-053039
E2_166cm	E2	166	Twig	2505	27	717	2514	2727	2491	2597							YAUT-073631
E4_113cm	E4	113	Seed	2077	25	2097	1994	2117	1947	2039							YAUT-079837
E4_115cm	E4	115	Stem?	1187	22	1172	1065	1179	1013	1110							YAUT-073638
E5_120cm	E5	120	Seed	2064	22	2052	1949	2107	1942	2021							YAUT-073632
E5_138cm	E5	138	Wood fragment	2161	28	2299	2102	2305	2008	2180							YAUT-073633
E6_112cm	E6	112	Bark?	992	21	952	830	957	798	886							YAUT-073639
E6_113cm	E6	113	Wood fragment	2166	26	2299	2113	2305	2054	2192							YAUT-079838

### Diatom assemblage analysis

Diatom assemblages were employed for reconstructing paleoenvironments and evaluating paleo-sea levels, as well as assessing the preservability of event layers. In the process of reconstructing the paleo-depositional environment, we examined modern diatom assemblages found in the river sand, river mouth mud, and low-surface sediments (Table 2). It is worth noting that tidal mud was not prevalent in the intertidal zone due to the present sandy beach conditions, which resulted in a limited presence of diatom valves. The lower-reach sand of the Kabari River and river mouth mud showed similar assemblages, including brackish diatoms such as *Navicula lanceolata* and *Navicula gregaria*. Freshwater species were dominated by epontic species such as *Achnanthes lanceolata*, *Fragilaria recapitellata*, and *Gomphonema* spp. In addition, freshwater benthic *Nitzschia* spp. were abundant in the river mouth mud. The low-surface sediments contained a few fresh-brackish water species, such as *Navicula eidrigiana* and *Rhopalodia gibba*, but most were freshwater benthic species such as *Caloneis* and *Pinnularia* spp. The proportion of Neogene diatoms was negligible in these reference sediments. To confirm whether the Neogene sedimentary rocks contain diatoms, sedimentary rocks were sampled at site D4 (Fig 1C), and Neogene diatoms were identified.

**Table 2:** Diatom assemblages of current references (%).

		River sand (D1)	River mouth mud (D2)	Low surface mud (D3)	Neogene rock (D4)
*Navicula gregaria*	*B	4	10	0	0
*Navicula lanceolata*	B	20	4	0	0
*Navicula eidrigiana*	*B-F	0	0	3	0
*Rhopalodia gibba*	B-F	0	0	4	0
*Achnanthes lanceolata*	*F	3	6	2	0
*Caloneis branderii*	F	0	0	3	0
*Caloneis leptosoma*	F	0	0	3	0
*Caloneis silicula*	F	0	0	3	0
*Encyonema silesacum*	F	10	17	2	0
*Fragilaria recapitellata*	F	16	12	1	0
*Gomphonema olivaceum*	F	6	0	0	0
*Gomphonema parvulum*	F	1	3	10	0
*Meridion circulare*	F	2	0	0	0
*Meridion constrictum*	F	2	2	2	0
*Nitzschia amphibia*	F	0	0	4	0
*Nitzschia dissipata*	F	0	4	0	0
*Nitzschia inconspicua*	F	6	9	0	0
*Nitzschia linearis*	F	1	4	0	0
*Nitzschia palea*	F	3	6	0	0
*Pinnularia subnodosa*	F	0	0	4	0
*Reimeria sinuata*	F	9	5	0	0
*Stauroneis anceps*	F	0	0	3	0
*Surirella angusta*	F	1	2	3	0
Neogene spp.		2	0	2	98
< 3% spp.		15	17	50	2

*B: Brackish water species

*B-F: Brackish to freshwater species

*F: Freshwater species

#### Sites E1–E3 in the east area

**Fig 3:**
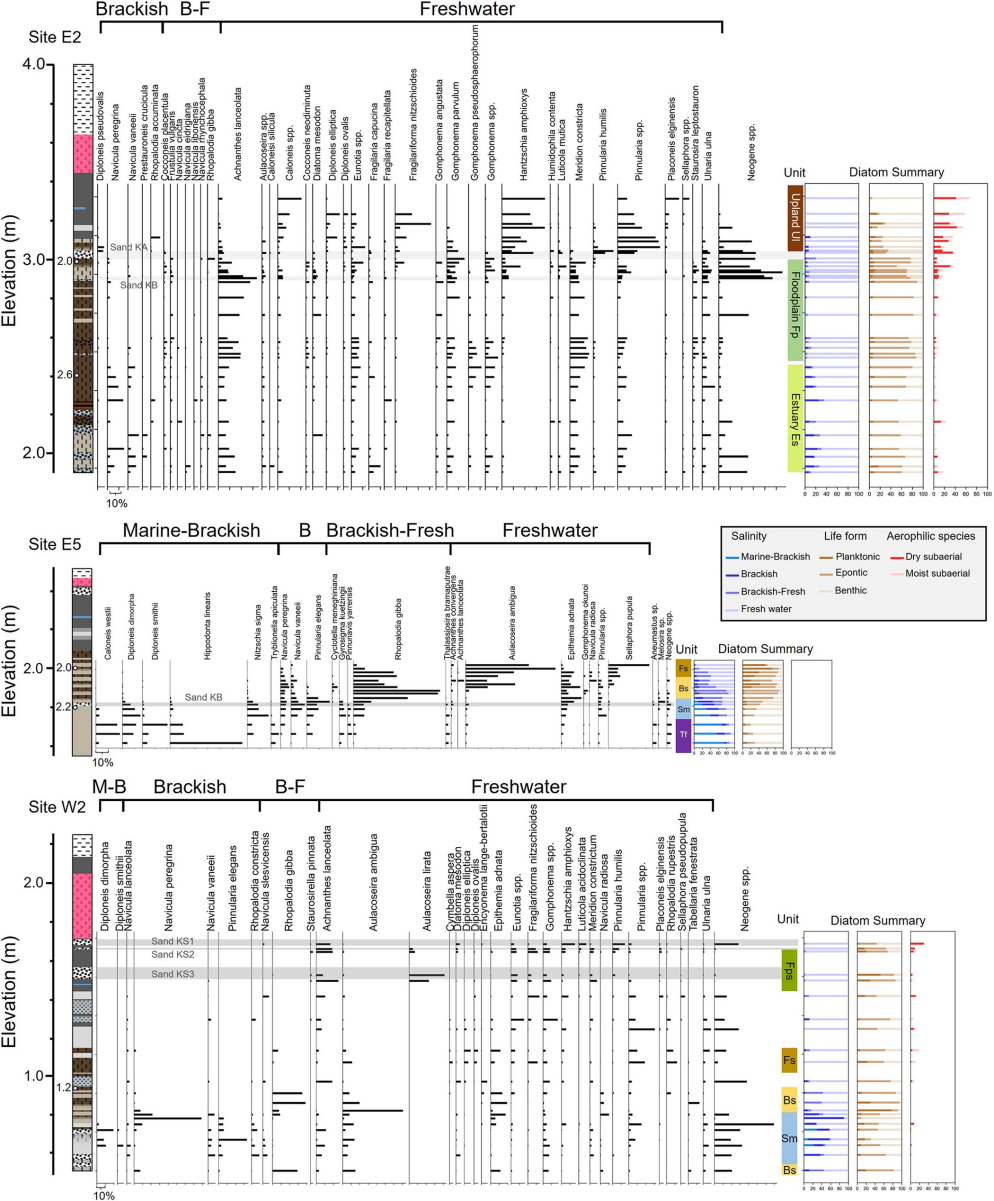
Diatom assemblage analysis results and interpreted depositional environment units. Abbreviations indicate M-B: Marine to Brackish, B: Blackish, B-F: Brackish to Freshwater species. The circles in geological columns show calibrated radiocarbon dates (ka).

We categorized into three distinct units: Unit Es (Estuary), Fp (Floodplain), and Ul (Upland), based on the diatom assemblages (Fig 3 and S2 Fig). Unit Es (<2.5 m asl) consisted primarily of inorganic gray silt and fibrous peat layers. This unit exhibited an abundance of approximately 30% of benthic brackish water species, such as *Navicula peregrina* and *Navicula vaneeii*. Additionally, *A*. *lanceolata*, *Gomphonema* spp., and *Meridion constricta* were dominant. Unit Fp (2.5–3.0 m asl) featured a fibrous peat layer and a notable increase in the proportion of *A*. *lanceolata*, *Gomphonema* spp., and *M*. *constricta*, along with a reduction in brackish water species. Unit Ul (>3.0 m asl) was characterized by a black peat layer. In this unit, the presence of rheophilic species decreased, while freshwater benthic species such as *Pinnularia* spp. and *Hantzschia amphioxys* dominated, accompanied by *Fragilariforma nitzschioides*, *Diploneis elliptica*, and *Caloneis* spp.

#### Sites E4–E6 in the east area

Based on the diatom assemblages, the units were divided into five groups named Unit Tf (Tidal flat), Sm (Saltmarsh), Bs (Blackish swamp), Fs (Freshwater swamp), and Fp (Fig 3 and S2 Fig). Unit Tf featured a dominance of brackish water species such as *Diploneis smithii*, *Hippondona linearis*, *Nitzschia sigma*, and *Tryblionella apiculata*. Unit Sm was abundant in *N*. *peregrina*, *Pinnunavis elegans*, and *Gyrosigma kuetzingii*. Unit Bs was characterized by the prevalence of planktonic species that adhere to aquatic plants, such as *R*. *gibba*, *Epithemia adnata*, and *Aulacoseira ambigua*, including brackish species such as *Cyclotella meneghiniana* and *N*. *peregrina*. Similarly, Unit Fs was dominated by *A*. *ambigua*, *R*. *gibba*, and *E*. *adnata*, as Unit Bs, but with a notable absence of brackish-water species. The freshwater benthic species *Sellaphora pupula* increased as *R*. *gibba* and *E*. *adnata* decreased toward the upper part. Unit Fp was observed at Site E6, including *A*. *lanceolata*, *Gomphonema* spp., and dry-tolerant species such as *D*. *elliptica* and *H*. *amphioxys* (S2 Fig).

#### Sites E7–E8 in the east area

The lower sandy sediments were dominated by rheophilic species with small amounts of saltmarsh and muddy tidal flat species, with the highest proportion of Neogene species (S2 Fig). The upper black peat layer showed assemblages similar to those of the low-surface sediments as the reference (Table 2).

#### Site W2 in the west area

Unit Sm transitioned from a low-marsh assemblage, where *Diploneis dimorpha* dominated, to a high-marsh assemblage primarily characterized by the dominance of *N*. *peregrina* as observed from the lower to the upper part of the unit (Fig 3). Additionally, this unit displayed a notable presence of Neogene species. In Unit Bs, there were few brackish species, and *R*. *gibba* and *E*. *adnata* dominated, along with the observation of the freshwater planktonic species *A*. *ambigua*. Unit Fs contained *A*. *ambigua* and *E*. *adnata*, but fresh-brackish water species were almost absent. The black peat above the B-Tm tephra was characterized by the freshwater planktonic species *Aulacoseira lirata*, and this section was named Unit Fps (Flood plain to Freshwater swamp). Unit Fps included rheophilic species such as *A*. *lanceolata*, *Gomphonema* spp. as well as Unit Fp. In the clay and silt layers (1.1–1.4 m asl), the Neogene species are highly abundant, followed by the rheophilic species such as *A*. *lanceolata* and *Gomphonema* spp.

### Features of the sand layers

**Fig 4:**
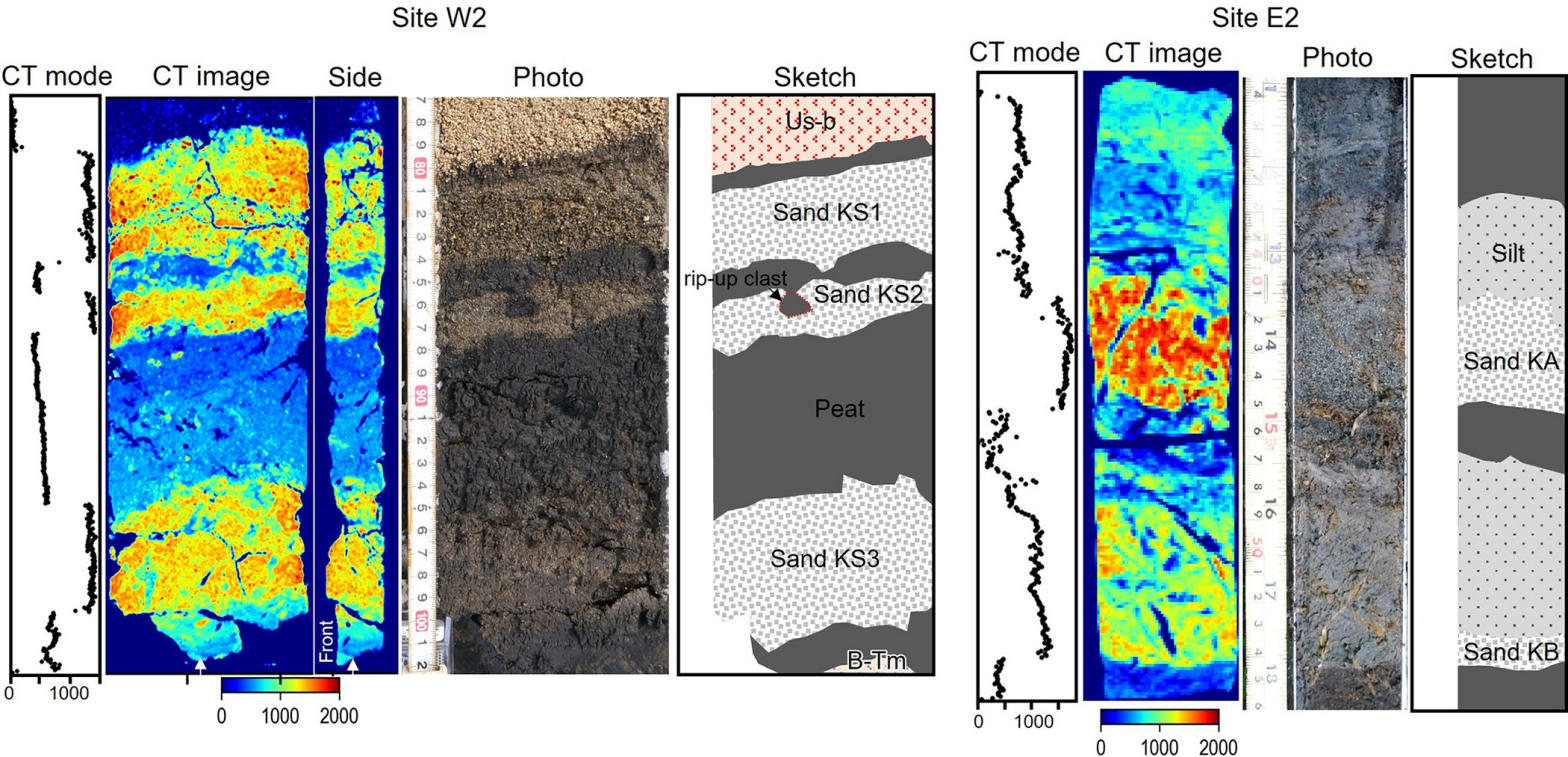
Core photographs and X-ray CT images at Sites W2 and E2. The CT mode profiles are plotted in correspondence with the CT images. For Site W2, a CT side image is shown, and its cross-sectional location is indicated by the arrows. The sketches display the interpretation of sedimentary structures of the sand layers and tephra layers.

**Fig 5:**
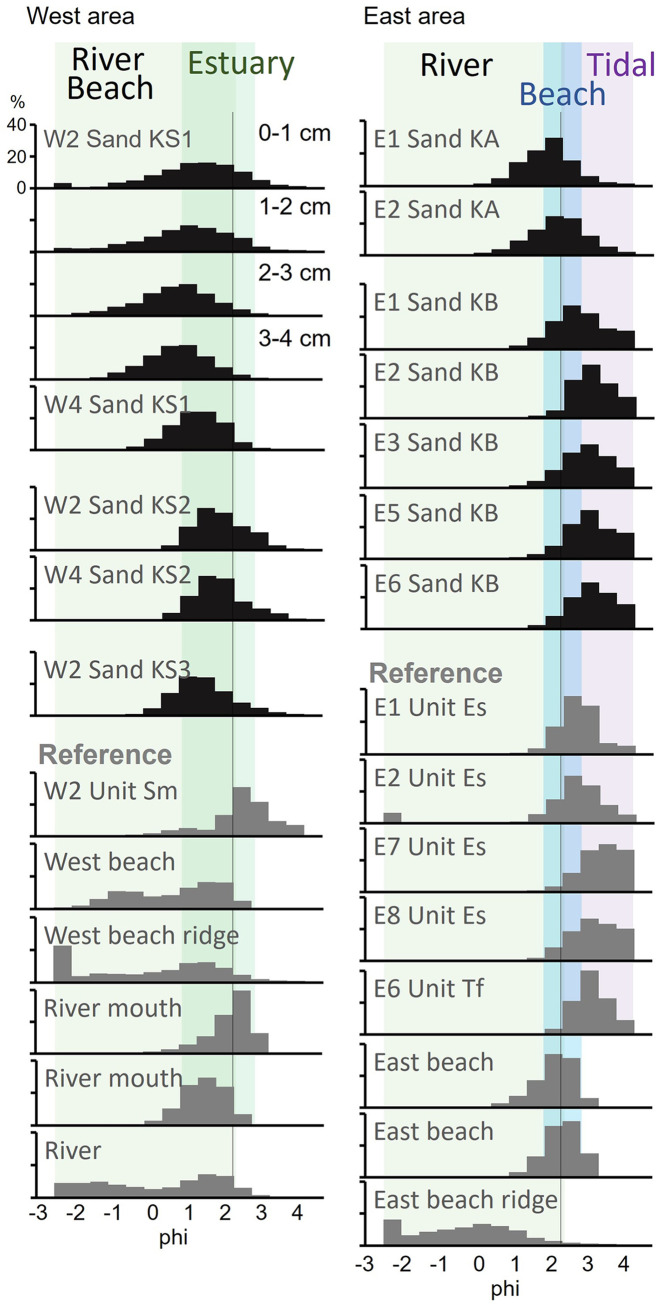
Grain-size distributions of the sand layers and reference sands of potential sources.

**Fig 6:**
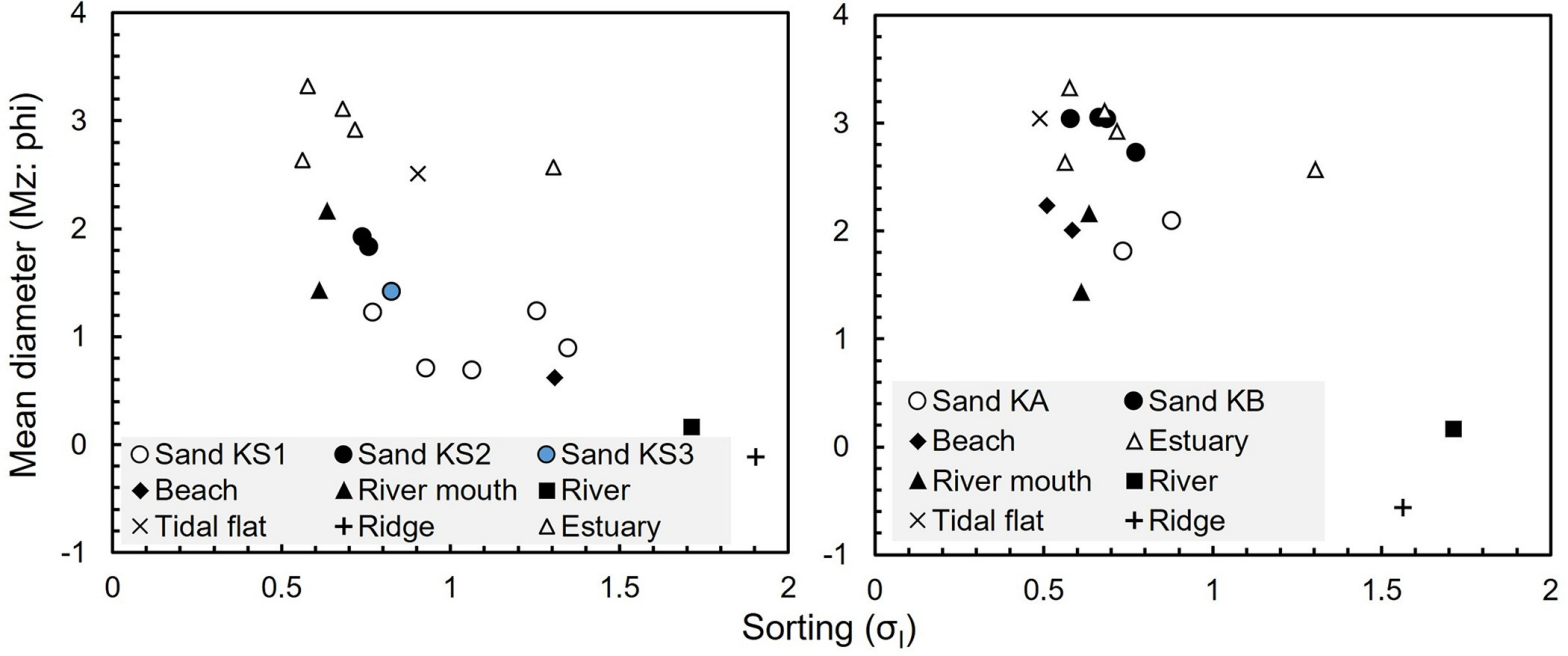
Biplots of sorting and mean diameter on grain size distribution. The left and right figures indicate the west area and the east area, respectively.

The depositional structures, thickness distributions, grain-size distributions, and contained diatoms were described to clarify the origin of the event layers (Figs 1C and 4–6). We targeted Sands KS1–KS3, KA, and KB as the event layers, which have clear contact with the underlying peats. The maximum thickness of Sand KS1 was 10 cm near the river mouth, thinning inland and becoming invisible 150 m (3 m asl) from the current coastline (Fig 1C). The sedimentary structure exhibited inverse- to normal-grading structures in the thick layer (Fig 5). The clear contact between the overlying and underlying black peats was accompanied by the observation of rip-up clasts (Fig 4). The grain-size distribution of reference sands in the east area could be roughly categorized into coarse-grained sands with poor sorting, including river, beach, and beach ridge sands, and fine-grained sands with well sorting found in river mouth sands. Sand KS1 showed the grain-size distribution that was located between that of the west beach and river mouth sands (Fig 6). Sand KS1’s diatom assemblages contained a notable 30% of Neogene species, with smaller proportions present in Sands KS2 and KS3, on the other hand, they were absent in the underlying peat layers.

Sand KS2 was separated from Sand KS1 by the peat layer with a few millimeters to 1 cm thick based on the CT image observation (Fig 4), exhibiting a time gap between their deposition. The distribution of Sand KS2 was more limited than Sand KS1 near the river mouth, at ~100 m (2.6 m asl) from the current coastline (Fig 1C). This layer was several millimeters thick (2 cm at the thickest). The contact between the underlying black peat and KS2 was clear, where rip-up clasts were observed (Fig 4). The grain-size distribution was similar to that of the river mouth sands (Figs 5 and 6).

Sand KS3 was exclusively identified at Site W2, the lowest boring site situated at 1.5 m asl. This sand layer exhibited an inverse-to-normal grading structure and displayed a grain-size distribution similar to river mouth sand (Figs 5 and 6). The diatom valves found within the sand layers at Site W2 closely resembled those in the underlying peat layer (Fig 3).

At Site E2, Sand KA had a layer thickness of approximately 5 cm. The layer displayed a sharp contact with the underlying peat (Fig 4). It showed a normal grading structure with a light brown silt layer overlying the sand layer. The predominant grain-size distribution consisted of medium sand, and it exhibited a poorly sorted distribution (Fig 5).

Sand KB was a thin sand layer, approximately 1 cm thick, and it featured a distinct and sharp contact with the underlying peat layers (as shown in Fig 4). In the upper section of this layer, a light brown silt layer with a clear normal-grading structure was observed. The grain-size distribution in this layer primarily comprised very fine sand with well sorting, displaying an intermediate distribution between estuarine or beach and tidal flat sands (Fig 6).

**Fig 7:**
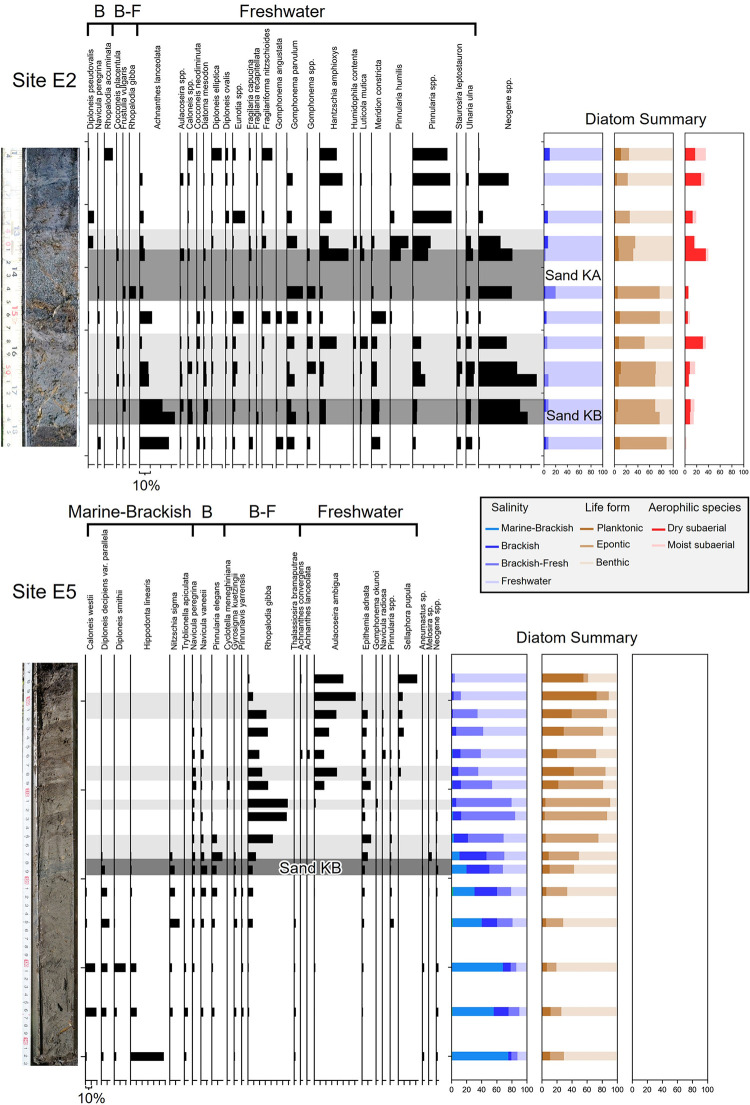
Diatom assemblages and close-up photographs near Sands KA and KB. Dark gray and light gray boxes indicate sand layers and silt layers, respectively.

For the silt layers overlying the sand layers, the CT mode profiles showed continuous changes between the sand layer and the overlying peat (Fig 4). The sand and overlying silt layers characteristically contained several to 25% Neogene species, which are rarely included in the underlying peat layers, with a decreasing percentage toward the overlying peats (Fig 7). Except for the Neogene species, these sand layers and overlying silts showed no significant change in diatom assemblages compared to the underlying and overlying peats.

## Discussion

### Reconstruction of depositional environments

**Fig 8:**
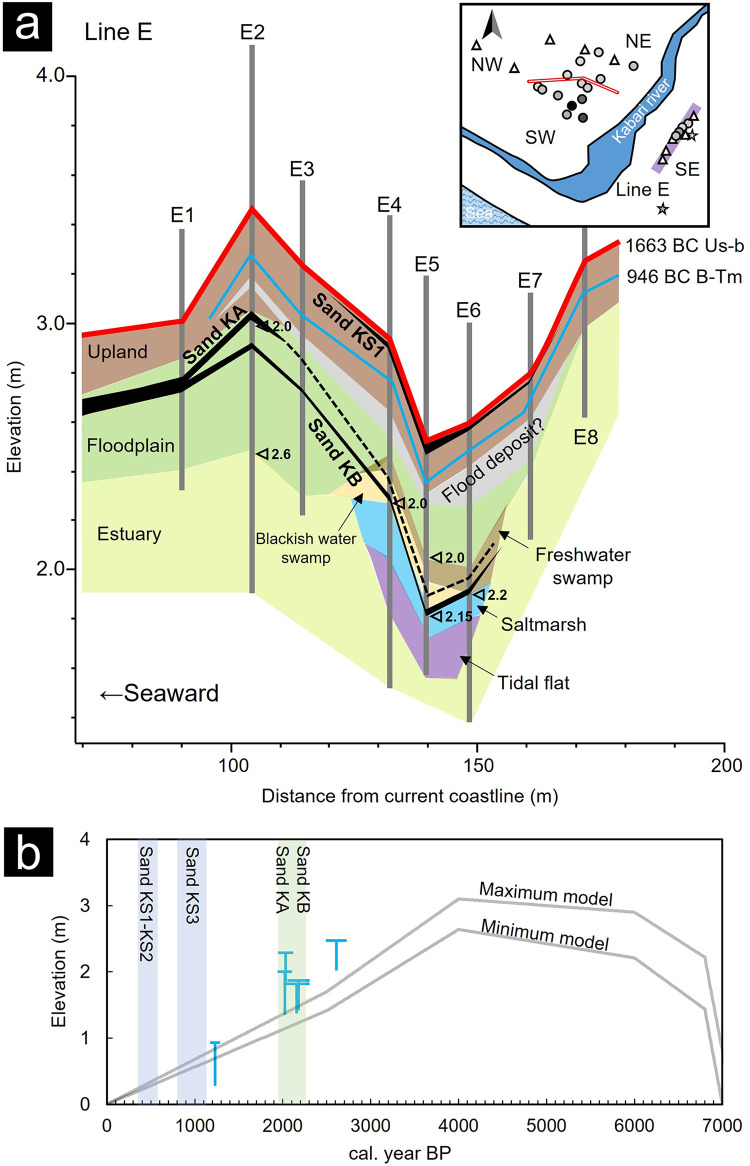
Reconstructed depositional environments and relative sea-level curves in the Kabari area. (a) Interpretation of the depositional environments along Line E is summarized from diatom assemblage analysis. (b) The results of SLIPs obtained in the Kabari area are summarized. The width and height of the T-shape symbols indicate the dating error and the altitude difference between SLIPs and mean sea level as tidal change range, respectively. The solid gray lines show the relative sea-level change curves, which are a combination of the local crustal deformation [26] and the GIA model obtained for the Shimokita Peninsula [58].

We discuss the depositional processes within this area, drawing insights from the diatom assemblage analyses and radiocarbon dating (Fig 8). To ensure precise age control, we relied on data obtained from selected samples. It is worth noting that bulk samples can yield misleadingly older ages, particularly when they incorporate older redeposited materials or plant fragments containing underground stems or roots with younger dating ages [55–57]. The ages were measured in equivalent stratigraphic intervals (E4_113cm and E4_115cm, E6_112cm, and E6_113cm, as detailed in Table 1 and Fig 2). In some cases, these ages exhibited discrepancies of over 1000 years. The results indicated that younger ages were inconsistent with the stratigraphic relationship with the B-Tm layer (Fig 2). These samples, although derived from large plant fragments, likely contained plant roots or stems from upper layers.

At Sites E1–E3, Unit Es exhibited a contribution from seawater until ~2.5 ka, with brackish species accounting for >20%. Unit Es indicated an estuarine environment because the indicator species for the middle to lower reaches of rivers were contained [32, 40] (Fig 3). Unit Fp showed assemblages similar to those of the low surface sediments and the dominant freshwater rheophilic species, which was interpreted as a floodplain deposit owing to sea level fall (Fig 3 and Table 2). In Unit Ul (after 2.0 ka), the groundwater table fell, as indicated by the increase in the proportion of the dry-tolerant species such as *H*. *amphioxys* and *Caloneis* spp. [32], and these sites changed to the present upland. At inland Sites E7 and E8, sandy sediments were deposited in an estuarine environment where allochthonous diatoms were transported from diverse environments (rivers and tidal flats) and changed to freshwater environments by sea level fall.

At Sites E4–E6, tidal flats were distributed until ~2.2 ka because *D*. *smithii*, *H*. *linearis*, *N*. *sigma*, and *T*. *apiculata* are indicator species of muddy tidal flats. As sea level fell, these sites transitioned into saltmarshes, characterized by the dominance of blackish water species typically found in low to high marshes [33]. In Unit Bs, these sites evolved into closed brackish swamps populated with waterweeds, given the presence of brackish and epontic or planktonic species. Unit Fs, on the other hand, exhibited few brackish-freshwater species and no significant change in other species composition, indicating a sustained closed environment that had shifted towards a freshwater environment. For Site W2 in the west area, a similar transition occurred from a saltmarsh to a closed swamp, mirroring the developments seen in Sites E4–E6, until ~1.2 ka (Fig 3). The environment subsequently shifted from a relatively tranquil freshwater swamp with planktonic species to a floodplain environment dominated by inorganic mud, demonstrating signs of redeposition and featuring freshwater rheophilic species. After B-Tm deposition, it changed again from a floodplain to a closed swamp environment dominated by the planktonic *A*. *lirata*.

In summary, the assemblage analysis results show that the estuarine environment was widely distributed in the east area until ~2.6 ka, with local tidal flats and saltmarshes developing around Sites E4–E6. Sites E1–E3 and E4–E6 changed to floodplain and closed swamp environments, respectively, until ~2.0 ka. After 2.0 ka, Sites E1–E3 and E4–E6 changed to upland and floodplain environments, respectively. In the middle surface of the west area, the estuary or saltmarsh continued until ~1.2 ka, transitioned to a floodplain until ~1.0 ka, and then from a closed swamp to an upland environment. The contribution of saltwater in the west area lasted longer than in the east area due to the westward tilt due to long-term crustal deformation.

The open environments such as estuarine and saltmarsh contain a high percentage of Neogene species. Neogene diatom valves are rarely found in the modern river, river mouth, and floodplain sediments (Table 2), making it unreasonable to attribute a high percentage from fluvial sources. The abundance of Neogene diatoms in the units (Tf and Sm) of open environments with the sea suggests that most fossils were induced from the sea-facing cliffs of Neogene sedimentary rocks by marine erosion (Table 2 and Fig 1C). The black peats contained few Neogene species, which also indicates that there were few fossil species at times close to the present, which have ceased marine erosion due to the sea regression and decreased their supply. The inorganic mud layers were identified throughout the area (e.g., 1.0 m asl and 1.1–1.4 m asl in W2, below B-Tm in Line E); however, these consisted of freshwater rheophilic species, dry-tolerant species, and Neogene species, which may be flood deposits from stormy weather.

### Estimation of relative sea-level change

Considering the global GIA, the local uplift of the 125-ka timescale for the Kabari area is 40–50 m [26, 29], with an average uplift rate ranging from 0.33–0.40 mm/yr. Applying the GIA model obtained for the Shimokita Peninsula [58], which is less affected by local crustal deformation, to the uplift in this area, a maximum sea level of + 3 m was obtained at 6–4 ka. The SLIPs of the Kabari area obtained from the diatom assemblage analysis were compared with the relative sea-level changes estimated with the GIA model and the local uplift trend (Fig 8 and Table 3). The obtained SLIPs were consistent with the estimated mean sea level of ~2.0 ka. However, the SLIPs at 2.6 and 1.2 ka indicated a more abrupt change in sea levels, suggesting a sea level curve with a larger slope.

**Table 3:** Summary of the sea level index point dataset in the Kabari area.

Site	Elevation	Material	2σ age (cal. yr BP)		Depositional environment	Indicative tide	Reference	Evidence
	(m asl)	dated	mean	age error			water level	
W2	0.93	Seed	1232	±56	Supratidal/Freshwater marsh	HAT	MSL + 0.65 m	*Epithemia adona*, *Rhopalodia gibba*
E2	2.47	Twig	2609	±118	Highmarsh/Supratidal	MHHW	MSL + 0.45 m	*Navicula peregrina*, *Navicura vaneeii*
E4	2.29	Seed	2032	±85	Highmarsh/Supratidal	MHHW	MSL + 0.45 m	*Navicula peregrina*, *Navicura vaneeii*, *Nitzschia sigma*
E5	1.82	Wood fragment	2157	±148	Highmarsh	MHHW	MSL + 0.45 m	*Navicula vaneeii*, *Nitzschia sigma*, *Pinnularia elegans*
E5	2.00	Seed	2025	±82	Supratidal/Freshwater marsh	HAT	MSL + 0.65 m	*Epithemia adona*, *Navicula peregrina*, *Rhopalodia gibba*
E6	1.87	Wood fragment	2180	±125	Highmarsh/Supratidal	MHHW	MSL + 0.45 m	*Navicula peregrina*, *Nitzschia sigma*, *Rhopalodia constricta*

### Formation factor of sand layers

In this area, five sand layers with sharp contact with peat layers were identified. The formation factors of these sand layers were discussed based on their sedimentary features, components, and distribution.

Sand KS1 was distributed >200 m inland considering the coastal erosion in the 1950’s. The distribution height of Sand KS1 was ~3 m asl, and wider and higher than that of Sand KS2 in the west area. In the east area, only one layer was identified just below the Us-b pumice in this study and Takashimizu et al. [11] (3.8 m asl: Fig 2). The distribution of this layer in the east area was higher than that of Sand KS2 in the west area. Therefore, we estimated that Sand KS1 was also correlated with the sand layer in the east area, or two sand layers may be amalgamated in a certain site due to near-time events. Sand KS1 showed a thinning trend toward the inland direction, an inverse-to-normal grading structure, and clear contact with peat. The averaged grain-size distribution showed a composition closest to that of the river mouth sands, and the coarse-grained components (<0.5 phis) suggest that it was supplied from a certain amount of beach and beach ridge sand or river sand (Fig 6). Based on the thinning distribution inland, supply from the upstream side of the Kabari River is unlikely. It is reasonable that this grain-size distribution results from mixing the sands of the river mouth and beach, suggesting that the coarse-grained beach sand was directly inundated beyond a berm. In addition, the abundance of Neogene species in this layer may suggest significant erosion of the terrace cliffs and their reworked deposits. The thickness distribution and grain-size distribution of this sand layer suggested that it was transported from the sea or the river mouth (Fig 1C). However, its diatom assemblage did not contain marine to brackish water species (Fig 3). This is due to the depositional environment in the flow path. Few valves of marine to brackish species at inland sites have often been reported in modern events [59–61]. Diatom abundance in marine and sandy beaches is estimated to be much smaller than in muddy terrestrial sediments because they prefer muddy sediments where colonization is possible and suffer a sorting on a grain size due to their small size. In this area, beaches or river mouths were the inundation pathways; however, muddy sediments are limited to these areas. Therefore, the diatom assemblage in the sand layer was similar to that of the underlying peat.

Sand KS2 was found only in the west area, with a fan-shaped distribution from the river mouth (Fig 1C), similar to that of Sand KS1, suggesting that it was transported by flows from the sea. Although the thin layer made it difficult to observe sedimentary structures, traces of erosion were observed, such as rip-up clasts and Neogene diatom valves. The lack of coarse-grained components in the beach ridge suggests that the inundation could not have exceeded a berm and transported fine-grained components from near the river mouth.

Sand KS3 was exclusively identified at Site W2, the lowest coring site (1.5 m asl). This sand layer exhibited an inverse- to normal-grading structure, and its grain-size distribution closely resembled that of river mouth sands (Fig 6), suggesting a potential origin from the river mouth. Although Sand KS3 exhibited similar features to Sand KS2 or Sand KS1, its relatively low elevation distribution makes it challenging to distinguish from storm deposits [62, 63].

Nakanishi and Ashi [25] previously developed models to understand sediment transport driven by tsunamis and typhoons in Shizunai, located along the central Hidaka coast. While Hokkaido is not frequently affected by large typhoons due to its location far from the equator, the estimated sand distribution around the river mouth, even under the impact of the most significant typhoon ever recorded in Hokkaido, reached altitudes of up to 1 m asl. Even considering the largest typhoons in Japan, which is an unrealistic scenario for Hokkaido, the predicted sand distributions reached altitudes of up to 2 m asl, albeit limited to the vicinity of the river mouth. While specific calculations for the Kahari area are necessary, Sands KS1 and KS2 exceed elevations of 2 m asl, surpassing the assumed limit for storm deposits. Given that Sand KS3 represents an older deposit, indicating a higher sea level at the time, it becomes challenging to entirely rule out the possibility of a storm deposit.

Turning to Sands KA and KB, we examined core-to-core correlations of these sand layers using radiocarbon dating, stratigraphy, and grain-size distribution. Despite differences in layer thickness and grain-size distribution (Fig 5), both exhibited pronounced normal grading structures covered by silt layers and featured sharp contacts with the underlying peat (Fig 4). Since the distance between cores along Line E was less than 15 m (Fig 8), it was feasible to distinguish Sands KA and KB based on their distinct grain-size distributions (Fig 6). At Site E1, two sand layers were identified, with the lower part comprising very fine sand and the upper part being medium sand, correlated to Sands KA and KB, respectively. Sites E3, E5, and E6 exhibited a sequence of very fine sand overlain by light-brown silt, consistent with Sand KB and the overlying silt, as evidenced by similarities in the grain-size distribution (Figs 2, 5 and 8). At Site E4, although no sand layer was observed, the silt layer may correspond to Sand KB based on dating results (Fig 2). Sand KA could potentially be correlated with silt layers above Sand KB at Sites E3–E6. Sand KB’s correlation extended from Site E1 to Site E6, covering up to 150 m inland from the current coastline. However, the thin sand layer may have been distributed slightly further inland, as the more inland Sites E7–E8 were not conducive to preserving event layers, such as estuaries and upland environments [22]. Sands KA and KB could be differentiated from the river sand because their grain-size distributions were consistent with those found from the east beach to the river mouth and tidal flat, respectively (Fig 6). The sea level at the time of Sands KA and KB deposition (~2.0 ka) was estimated to have been at least 1 m higher than the present sea level (Fig 8). After subtracting the estimated sea level, the distribution altitude of these sand layers was ~2 m.

Clear contact with the underlying peat and a set of inorganic mud layers covering the sand layer is indicative of abrupt crustal subsidence or a mud cap found in tsunami deposits [64–67]. In the former case, a rapid change—from a moment to a few years—to a diatom assemblage dominated by brackish water species is expected [64–66]. In contrast, sediments transported by strong flows have diverse species assemblages, ranging from freshwater to marine species, as has been reported for modern tsunami deposits [59–61]. The diatom assemblages at Sites E2 and E5 showed few changes between the sand and overlying silt layers (Fig 7). The silt layers contained *H*. *amphioxys*, a dry-tolerant species, suggesting also its transport from inland areas. Sands KA and KB contained a small number of brackish species, which indicates their transport from seaward. The sand layers and overlying silts contained large amounts of Neogene diatoms, suggesting that the source of this silt layer was likely estuarine sediments (Unit Es) or partly from the Neogene sedimentary rocks. The CT mode displayed a continuous decreasing trend (Fig 4), suggesting that the sand and silt layers were continuously deposited as a single unit. The dating results obtained using bulk samples below and above the sand layers indicated that the overlying silty peats were older than the underlying fibrous peats (Fig 2), indicating that the overlying silty layers were redeposited due to the erosion of older organic matter [68]. These results indicate that the overlying silt layers were mud caps derived from reworked sediments without rapid environmental changes.

To conclude, the extensive sheet distribution and the single grading structure with the mud cap observed in Sands KS1, KS2, KA, and KB indicate a rapid single flow event [60–62, 67, 69]. This characteristic sets them apart from river flood sediments, typically exhibiting muddy sediments and poor sorting due to a prolonged flow [70, 71]. The fan-shaped distribution pattern and grain-size composition further suggest a source either from the beach or the river mouth. Moreover, the layer distributions exceed the predictions based on the numerical simulations of the largest storms in Hokkaido. Based on these interpretations, it is highly likely that at least four of the sand layers represent tsunami deposits.

### Wave source of tsunami deposits

**Fig 9:**
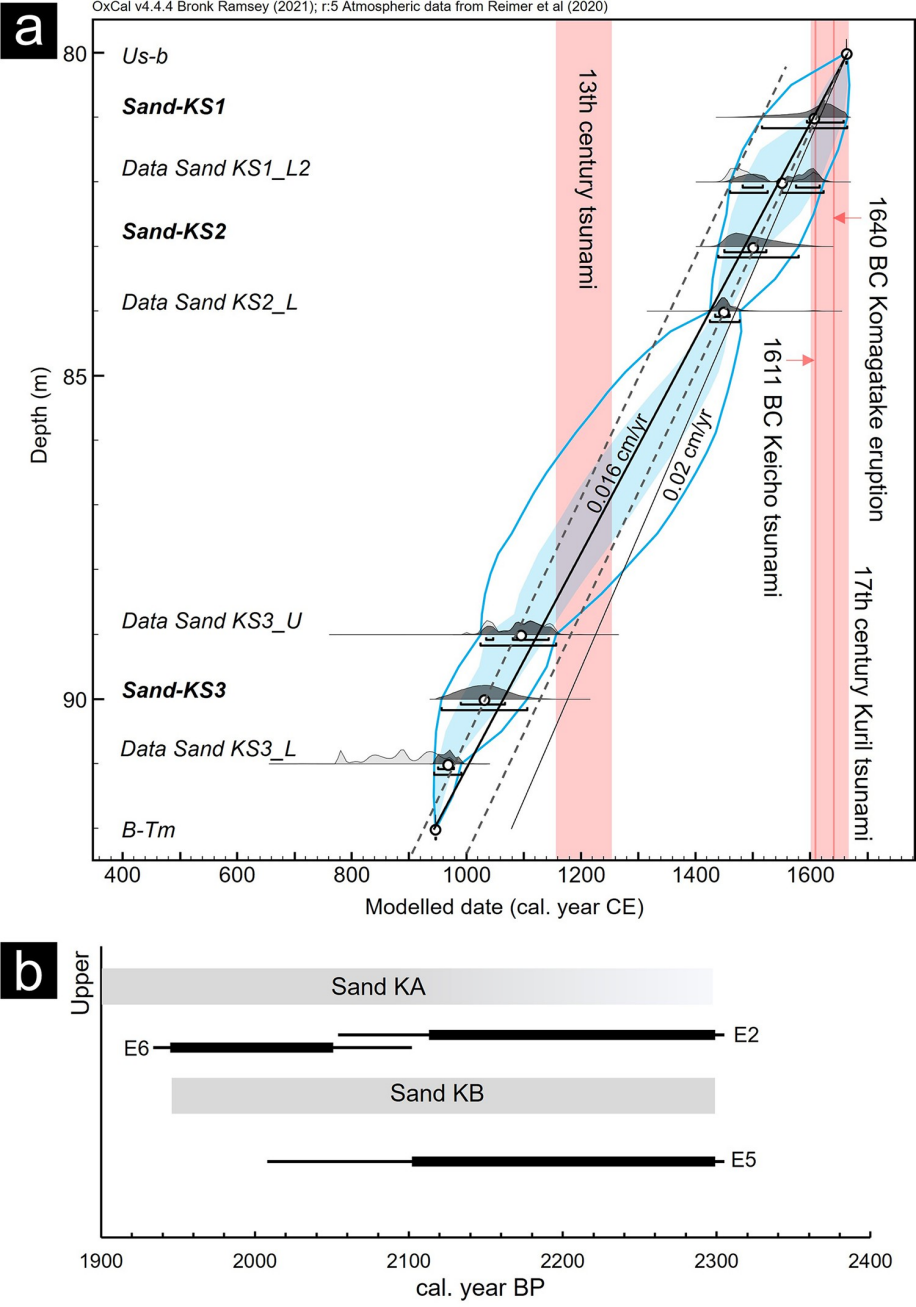
Depositional ages of the sand layers based on radiocarbon dating. (a) Age-depth model in Site W2. The light and dark gray histograms show the calibrated and modeled probability density functions, respectively. The blue solid lines and blue shade indicate the ranges of the 2σ and 1σ modeled ages, respectively. The red bands indicate the ages of the tsunami events reported [19] and the historical records. The dotted lines show the sedimentation rate trends of the upper (Us-b–Sand KS2_L) and lower strata (Sand KS3_U–B-Tm) based on the average values. The solid black lines show sedimentation rates of 0.016 cm/year and 0.020 cm/year as a reference. (b) The summary of the calibrated ages measured in the peat layers above and below Sand KA and Sand KB each boring site along survey line E. The thick and thin black lines indicate the ranges of the 2σ and 1σ calibrated ages, respectively. The gray bars show the estimated depositional ages of the sand layers.

The origin of the potential tsunami deposits was estimated by comparing the chronology of the tsunami events in the surrounding areas. The age of the stratigraphic interval from the Us-b to B-Tm tephras was constrained by the age-depth model using the P_sequence module (Fig 9). The results showed that the depositional ages with 2-sigma error of Sand KS1–KS3 were 1516–1663, 1425–1477, and 946–991 CE, respectively. However, these ages may be older than the actual depositional ages owing to several factors. First, the two lower samples were measured using the bulk samples. For the age gaps, a redeposition effect on the two samples was considered smaller than other dating results of bulk samples because it was a quiet depositional environment dominated by planktonic species such as *Aulacoseira* spp. (Fig 3). The age-depth model showed a trend in which the modeled age shifted toward an older age in the interval measured bulk samples. The actual depositional ages of the peat around the Sand KS3 layer were estimated to be younger by >100 years based on the sedimentation rate of the upper peat. Second, it is possible that the surface sediments were eroded at the time of the event and that the measured samples were older [56, 68]. Sand KS1 and KS2 indicated sharp contacts and rip-up clasts, suggesting an extreme flow caused erosion. Tsunami deposit studies of the 869 Jogan tsunami reported a gap of ~100 years [68]. Therefore, the actual depositional ages of the Sands KS1 and KS2 layers were assumed to be younger than the modeled ages. Similarly, Sand KS3 may be correlated with the 13th-century event in the Kuril Trench [2, 19], and Sand KS2 also may be correlated with the 17th-century events.

The depositional ages of Sands KA and KB were estimated from the dating results above and below the sand layers in the multiple cores (Fig 9). The ages of the peats above and below Sand KB were estimated to be 2.3–1.9 and 2.3–2.0 ka, respectively. In the southern Hidaka area, events of 2.3–2.1 and 1.9–1.8 ka were reported for Utoma and Erimo [2, 21] and are possible to be correlated with these tsunami deposits. However, the overlying silt layer makes it difficult to determine the upper boundary of the event layer, and as a result, the event ages are not well constrained. It is necessary to examine the event correlation using samples suitable for dating in the future.

If these sand layers indeed represent tsunami deposits resulting from megathrust earthquakes occurring at intervals of at least several hundred years, one might expect to find more tsunami deposits than the ones identified. The sand layers were deposited in enclosed environments such as back-barrier areas and freshwater to brackish water swamps (Figs 3 and 8), which are suitable for preserving event layers [22]. In some sites along Line E and Site W2, sand or silt layers were identified in stratigraphic intervals corresponding to higher sea levels in addition to the discussed sand layers. These sand and silt layers exhibited characteristics of muddy sand with poor sorting and indistinct sedimentary structures. Consequently, it’s possible that they were subsequently disturbed after temporary deposition by tsunamis, as sand layers are typically poorly preserved in saltmarsh or estuary environments due to bioturbation and running water (Figs 3 and 8). The depositional age of the event layers was limited to the past ~2200 years, leaving a gap of over 1000 years. For these reasons, sediment archives for event layers in this area appear incomplete, and the possibility of tsunamis occurring in depositional environments unsuitable for preservation cannot be ruled out.

The estimated depositional age of Sand KS1 aligns with historical records of the 17th-century tsunami in the Kuril Trench, the 1640 CE Komagatake tsunami, and the 1611 CE Keicho tsunami. Sand KS1 exhibited a broader and higher distribution (>3 m asl) compared to the other four layers (~2 m asl). Numerical simulations of the 1640 CE Komagatake collapse and the 17th-century Kuril earthquake models estimated wave heights of 4 and 2 m in the Kabari area, respectively [2, 12]. Moreover, this model’s results roughly correspond to the distribution heights of Sand KS1 and the other sand layers. Sand KS1, being the most widespread among the five events, may have resulted from an extraordinary event, such as the collapse of Mt. Komagatake. However, comprehensive research spanning different regions is required to determine the exact wave source, as there are variations in magnitudes and rupture zones even among earthquakes in the Kuril Trench with recurrence intervals of several hundred years [2, 8, 9]. Moreover, comparing the distribution of tsunami deposits with simulated inundation areas on a 2D topography will be crucial for future research. Additionally, it is necessary to consider factors like the presence of a nearby active fault and the 1611 CE Keicho tsunami or the northern Sanriku-oki earthquake facing the Hidaka coast.

## Conclusions

To assess the tsunami magnitude resulting from both the giant earthquakes in the Kuril Trench and the collapse of Mt. Komagatake, we conducted additional field surveys in the Kabari area of the northern Hidaka region. This area was expected to have been impacted by tsunamis from both sources. We identified five sand layers exhibiting features common to modern tsunami deposits based on layer distribution, grain size analysis, and sedimentary structures. Although the depositional ages of these sand layers have been biased within the last 3000 years due to limited suitable depositional environments for preservation, it seems certain that multiple tsunamis have reached this area during this timeframe. We compared the distribution altitude of tsunami deposits within the region by reconstructing paleo-sea levels during these events. Four sand layers exhibited similar elevations, approximately 2 m in height, except for the latest one. Most of these layers correlated chronologically with earthquakes occurring at intervals of several hundred years in the Kuril Trench. However, the latest sand layer in the 17th century displayed an unusual distribution, exceeding an elevation of 3 m. This suggests that it may have originated from a distinct tsunami source, possibly the collapse of Mt. Komagatake in 1640 CE. To verify this hypothesis, future efforts should focus on replicating the distribution of these sand layers using wave source models for megathrust earthquakes and the Mt. Komagatake collapse. The distribution and depositional age data of these sand layers offer a basis for comparing the magnitudes of the two tsunamis around the 17th century, providing valuable constraints for developing fault models in the future.

## Supporting information

S1 Fig Wide-area map of northern Hidaka and aerial photograph in 1944. (DOCX)

S2 Fig Summaries of diatom assemblage analysis and interpreted sedimentary environmental units of Site E1, E3, E4, and E6–E8. (DOCX)
