# Supplementary material for: Understanding paleo-earthquakes in the Kuril Trench based on Late-Holocene tsunami deposits in the distal region from wave sources, northern Hidaka, Hokkaido, Japan
Source: PLoS One. 2024 Apr 17;19(4):e0298720. doi: 10.1371/journal.pone.0298720 (PMC11023580; doi:10.1371/journal.pone.0298720)
Supplement: S1 Fig — (DOCX) [file pone.0298720.s001.docx]

Supplemental Information

Understanding paleo-earthquakes in the Kuril Trench based on Late-Holocene tsunami deposits in the distal region from wave sources, northern Hidaka, Hokkaido, Japan

Ryo Nakanishi, Juichiro Ashi, Satoshi Okamura, Yusuke Yokoyama, Yosuke Miyairi

Figures S1


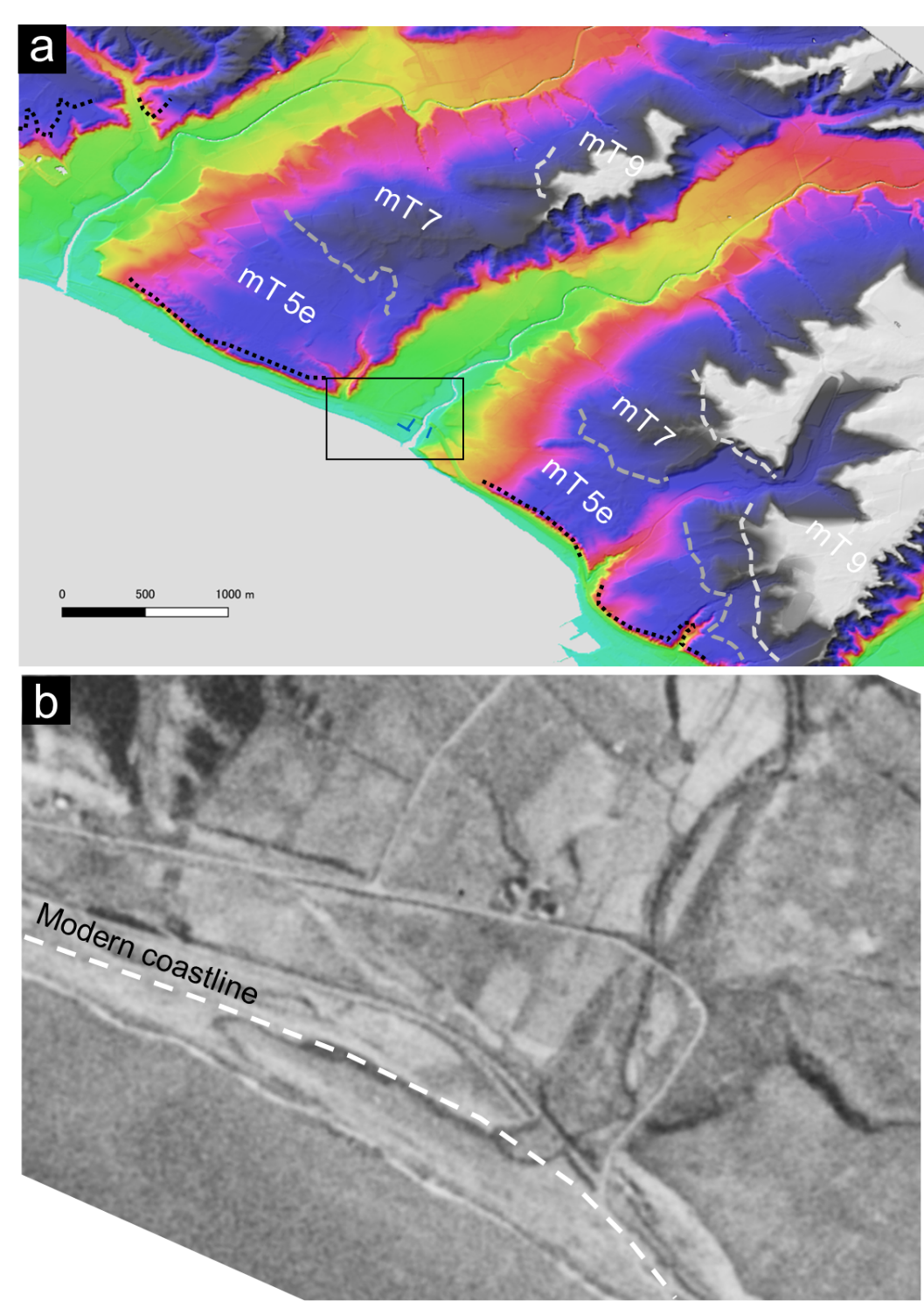


**S1 Fig. Wide-area map of northern Hidaka and aerial photograph in 1946.** a: Digital elevation model and distribution of marine terraces around the Kabari area. Blue lines show the survey lines. b: Aerial photograph of 1948 in the Kabari area. The digital elevation map and the aerial photographs provided by the Geospatial Information Authority of Japan.
