# Supplementary material for: Understanding paleo-earthquakes in the Kuril Trench based on Late-Holocene tsunami deposits in the distal region from wave sources, northern Hidaka, Hokkaido, Japan
Source: PLoS One. 2024 Apr 17;19(4):e0298720. doi: 10.1371/journal.pone.0298720 (PMC11023580; doi:10.1371/journal.pone.0298720)
Supplement: S2 Fig — (DOCX) [file pone.0298720.s002.docx]

Supplemental Information

Understanding paleo-earthquakes in the Kuril Trench based on Late-Holocene tsunami deposits in the distal region from wave sources, northern Hidaka, Hokkaido, Japan

Ryo Nakanishi, Juichiro Ashi, Satoshi Okamura, Yusuke Yokoyama, Yosuke Miyairi

Figures S2


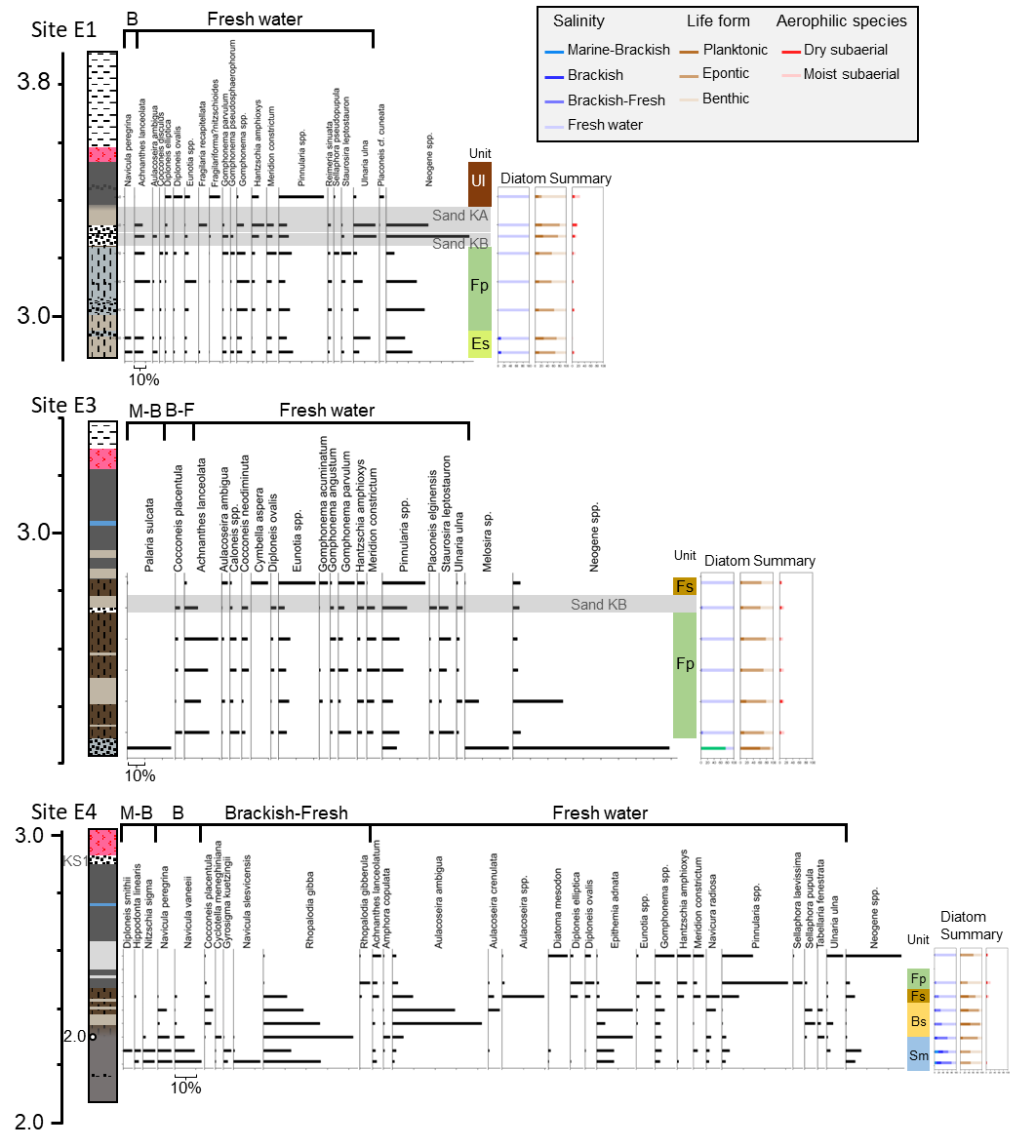


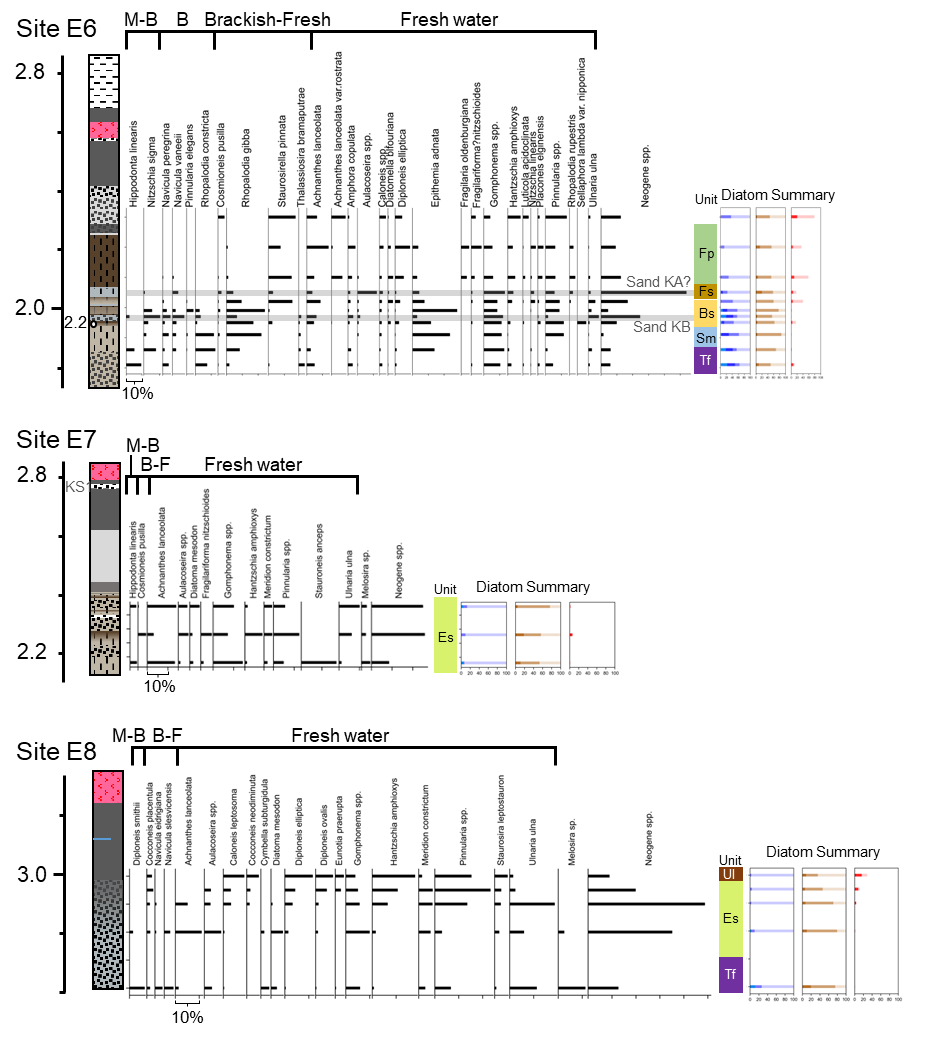


**S2 Fig. Summaries of diatom assemblage analysis and interpreted sedimentary environmental units.**
